# On the Proton Conduction Pathways in Polyelectrolyte Membranes Based on Syndiotactic-Polystyrene

**DOI:** 10.3390/membranes12020143

**Published:** 2022-01-24

**Authors:** Maria-Maddalena Schiavone, Yue Zhao, Hiroki Iwase, Hiroshi Arima-Osonoi, Shin-ichi Takata, Aurel Radulescu

**Affiliations:** 1 Jülich Centre for Neutron Science, Forschungszentrum Jülich GmbH, 85748 Garching, Germany; schiavonemariamaddalena@yahoo.it; 2 Department of Advanced Functional Materials Research, Takasaki Advanced Radiation Research Institute, National Institutes for Quantum Science and Technology (QST), Watanuki-machi 1233, Takasaki 370-1292, Gunma, Japan; zhao.yue@qst.go.jp; 3 Neutron Science and Technology Center, Comprehensive Research Organization for Science and Society (CROSS), 162-1 Shirakata, Tokai 319-1106, Ibaraki, Japan; h_iwase@cross.or.jp (H.I.); h_arima@cross.or.jp (H.A.-O.); 4 Materials and Life Science Division, Japan Proton Accelerator Research Complex (JPARC), Tokai 319-1195, Ibaraki, Japan; shinichi.takata@j-parc.jp

**Keywords:** proton exchange membranes, semi-crystalline polymers, small-angle neutron scattering

## Abstract

When functionalized by the solid-state sulfonation process, the amorphous regions of the semi-crystalline syndiotactic-polystyrene (sPS) become hydrophilic, and thus can conduct protons upon membrane hydration, which increases the interest in this material as a potential candidate for applications with proton exchange membranes. The resistance of sulfonated sPS to oxidative decomposition can be improved by doping the membrane with fullerenes. In previous work, we have described the morphology in hydrated sulfonated sPS films doped with fullerenes on different length scales as determined by small-angle neutron scattering (SANS) and the structural changes in such membranes as a function of the degree of hydration and temperature. In the current work, we report on the relationship between the morphology of hydrated domains as obtained by SANS and the proton conductivity in sulfonated sPS-fullerene composite membranes at different temperature and relative humidity (RH) conditions. Based on this combined experimental approach, clear evidence for the formation and evolution of the hydrated domains in functionalized sPS membranes has been provided and a better understanding of the hydration and conductivity pathways in this material has been obtained.

## 1. Introduction

Polymer electrolyte materials (PEM) for fuel cells applications (PEMFC) are characterized by a nanoscale phase separation into hydrophilic domains and hydrophobic regions, which is a combination that enables a high proton conductivity and provides a good chemical and mechanical stability, thus membrane durability. Despite its excellent conductive properties, the Nafion [1,2,3,4,5,6,7,8], which was established as the benchmark for such applications, presents several drawbacks related to the cost, safety, and supporting equipment during manufacturing and use [6]. Alternative low-cost semi-crystalline materials that present similar conductive and chemo-mechanical properties as the Nafion membranes are continuously searched for.

Syndiotactic polystyrene (sPS) is a stereoregular polymer that is easily crystallizable (typical degrees of crystallinity are in the 30–50% range) and presents very complex polymorphic behavior. Several crystalline phases with polymer chains arranged in either the trans-planar or helical conformation were identified and characterized [9,10,11], with the δ- and ε-phases, which are formed by crystallization from solution, representing co-crystals of s-PS with low molecular mass guest molecules (clathrates). This property offers the possibility to load different guests molecules in the cavities formed between the helices of the sPS in the crystalline regions by using the guest-exchange process [12,13], which makes the sPS suitable for different possible applications, such as fluorescent materials (with chromophore guest molecules), optical memories (with photo-reactive guest molecules), non-linear optical materials (with polar guests), and chiro-optical memories (with chiral guest molecules) [14]. On the other hand, given the recent developments, which enable a controlled sulfonation of only the amorphous phase, preserving thus the crystallinity of the material [15], and an improved resistance to oxidation decomposition when fullerenes are added [16], the sulfonated syndiotactic polystyrene (s-sPS) may become a good potential candidate for some PEMFC applications, as it presents high proton conductivity comparable to Nafion [17,18], high chemical and thermomechanical and chemical stability [16,19,20], and a low cost [21]. The preparation of an s-sPS membrane should start from the δ-form (clathrate with guest molecules), which enables a homogeneous sulfonation of only the amorphous regions and can be subsequently transformed into the thermodynamically stable β-form by high-temperature annealing procedures [17].

The proton conduction in PEMs depends on water and is governed by the water behavior at different length scales: at the molecular scale—dissociation of protons and formation of ion-pair with water, at the nanoscale—the transport through the hydrated domains, at the mesoscale—the long-range mobility within the water network. Therefore, in order to understand the transport properties in different conditions one should, first of all, understand the morphology of the hydrated domains at different length scales as a function of the hydration level and temperature and learn about the micro-dynamics in hydrated membranes under such conditions. In previous works [22,23,24], we reported detailed microstructural characterizations of sulfonated membranes based on the s-sPS δ-clathrates by extended Q-range small-angle neutron scattering (SANS, where Q = 4 π λ^−1^ sin(θ/2) is the modulus of the scattering vector $\vec{Q}$ [25], with λ the incident neutron wavelength and θ the scattering angle), complimented by WAXD, FTIR, UV-Vis, and TEM analyses. Membranes with different sulfonation degrees, with and without fullerenes added (by dipping the films in a fullerene-saturated toluene solution or by casting composite membranes from a common sPS/C60 solution in toluene, followed by sulfonation) were investigated in dry and hydrated states at room and elevated temperatures, up to 80 °C. The use of uniaxially deformed film samples, and neutron contrast variation allowed for the identification and characterization of different structural levels with sizes between nm and μm, which form and evolve with the variation of the hydration level and temperature. In all these studies, we focus mainly on the characterization of the morphologies formed in the amorphous functionalized regions of the material. The crystalline regions, which were loaded with different guest molecules in either the deuterated or protonated state, to provide the proper contrast for neutrons, were not affected by the sulfonation or the subsequent hydration or thermal treatment of the membranes, as demonstrated by the WAXD and SANS observations prior and after the membrane hydration and thermal treatment [22,23], including here the exposure of the membrane to Fenton’s test conditions [24]. Thus, the preservation of the membrane crystallinity, as it was demonstrated by the WAXD and the extended Q-range SANS observations, is a direct indication of the membrane robustness and stability during various hydration/temperature treatment procedures, which were meant to yield the hydrated and conductive morphologies in the amorphous regions of the materials. It is also worth noting that the membranes based on the crystalline δ-or β-forms of the sPS become hydrophilic by the functionalization (sulfonation) of the phenyl rings of sPS in the amorphous regions, and exhibit a proton conductivity comparable to that of Nafion, without any subsequent chemical treatment [15]. However, free radicals such as hydroxyl and hydroperoxyl are produced during the operation of the PEMFC as a result of the reaction of hydrogen and oxygen on the electrodes or the decomposition of hydrogen peroxide with metal contaminants in the membrane. These radicals initiate processes of chemical degradation that affect the durability and the lifetime of the PEM, as observed in the case of Nafion or other PEM materials [26,27]. Several approaches have been proposed to overcome this issue, among them to load carbon nanomaterials like CNTs, graphene oxide, or fullerenes in the PEM, as additives with radical scavenging properties [16,28,29]. Therefore, our interest is in studying the structure and properties of sPS-based PEMs with and without fullerenes incorporated in the membranes.

We report here on the common analysis of the results of the SANS investigation on the microstructure and of the measurement of the proton conductivity on a composite membrane made of s-sPS and C70 fullerenes. By comparison to the structural and proton conductivity results on fullerenes free s-sPS membranes, this combined approach provides direct evidence of the formation and development of the hydration domains in functionalized sPS membranes as a function of the degree of hydration and the temperature to which they are exposed and helps form a better understanding of the proton conduction pathways in these materials.

## 2. Materials and Methods

As-cast and uni-axially deformed sPS films were used in the previous structural investigations by SANS and for the proton conductivity measurements. All films were made of deuterated sPS (C_8_D_8_)_n_, which provides a low incoherent background in the neutron scattering experiments. Films containing fullerenes were prepared as composite membranes by casting from common sPS and C70 or C60 solution in toluene. Particularly for this study, a composite sPS-C70 membrane was prepared by casting from a common sPS and C70 toluene solution with 1 wt% C70 content. A second membrane with a C70 fullerene content in the initial solution of 3 wt% was prepared for a parallel pre-characterization regarding the condition of the fullerenes incorporated in the membrane.

Preparation and subsequent treatment—clathrate formation, sulfonation, guest-exchange in the crystalline region—of uni-axially deformed deuterated syndiotactic polystyrene films were described elsewhere [22]. The sulfonation of sPS/C70 membranes used in the current study followed the procedure described in [22]. For the SANS experiment, the exchange of the guest molecules in the polymer clathrate form from d-chloroform, which was loaded into the composite membrane during the sulfonation process, to d-toluene was achieved by immersing the films in the new solvent for 1 day, followed by drying at 40 °C under vacuum for a few hours. A detailed description of the preparation method of the composite membranes of s-sPS and fullerenes can be found in [24].

All reagents used for the preparation of the membranes, excepting the deuterated syndiotactic-polystyrene, were purchased from Sigma-Aldrich (Munich, Germany) and used as received. The deuterated syndiotactic-polystyrene was synthesized in collaboration with University “Federico II”, Naples, Italy, following the procedure which is described in detail in [22].

The investigation of the membrane microstructure by SANS was carried out on the time-of-flight (TOF) SANS diffractometer TAIKAN (BL-15), at the Material and Life Science Experimental Facility (MLF) of the Japan Proton Accelerator Research Facility (J-PARC), Tokai, Japan [30]. A Q-range between 0.005 and 1 Å^−1^ was covered in reciprocal-space [25], which corresponds to an investigation of structural sizes over a length scale between a few Å and 200 Å in the real space. Details of the methodical procedure and data analysis routines can be found in [22,23,24]. The temperature and humidity of the sample were controlled in a multi-position humidity sample chamber, which was designed and produced in house (Comprehensive Research Organization for Science and Society CROSS, Tokai, Japan), by using the so-called two-temperature method: dry air from a ga cylinder/compressor passes through the H_2_O in a tank and becomes pre-saturated vapors with a dew point almost equal to the water’s temperature (T_w_), then goes to the sample chamber which is at the temperature T_s_, with the RH being calculated from the saturated vapor pressure (*P*ws) at the dew point (T_w_) and the chamber temperature (T_s_).

Complementary analyses to SANS done by WAXD and membrane quality check by UV-Vis optical microscopy, thermo-gravimetric analysis (TGA), and prompt-gamma neutron activation analysis (PGAA) were carried out.

The incorporation of the fullerenes in the sPS-C70 composite membrane was checked by UV-Vis and thermo-gravimetric analysis (TGA). UV-Vis analysis was carried out at a Cary 100 SCAN UV-Vis Varian spectrometer (Agilent, Santa Clara, CA, USA) with the film samples placed in a specific holder equipped with quartz windows. The spectra were collected in the range 200–800 nm at a resolution of 100 nm/min. The TGA analysis was done on a TG 209 F1 Libra instrument—NETZSCH (NETSCH-Gerätebau GmbH, Selb, Germany) in the temperature range between 30 °C and 1000 °C at a heating rate of 5 °C/min with nitrogen flow at 60 mL/min.

Additional insight about the structure and morphology of C70 agglomerates in composite membranes were obtained by optical microscopy with a Leica DM6000 M light microscope (Leica Mikrosysteme Vertrieb GmbH, Wetzlar, Germany) in bright-field and crossed polarizers modes and by WAXD analysis of films by means of an X-ray powder diffractometer Brucker 2nd Gen-D2 Phaser (Cu-source) (Brucker, Karlsruhe, Germany).

The degree of sulfonation, expressed as SD atoms/styrene units × 100 mol% and further indicated as SD, was checked at the neutron prompt-gamma activation analysis (PGAA) instrument at the Heinz Maier-Leibnitz Zentrum (MLZ, Garching, Germany). Full descriptions of the experimental method and data interpretation can be found in [22].

The conductivity of the sPS-C70 composite membranes was measured in the plane direction at 100 kHz using a four-point probe alternating current electrochemical impedance spectroscopy (EIS) with an electrode system connected to an LCR meter (HIOKI 3522 LCR HiTESTER, Nagano, Japan). For the determination of the conductivity in liquid water, the membrane was equilibrated in H_2_O at 25 °C and 80 °C and placed between two platinum electrodes in air. For the measurement of the conductivity at different hydration levels from the vapor phase and different temperatures, the membrane was placed in a BT-115 Conductivity Cell (Scribner Associates, Southern Pines, North Carolina, USA) equilibrated by a temperature/humidity controller (HUM-1F, Rigaku Co., Tokyo, Japan) wherein the RH range was 10–80% at the prescribed temperature within the range 25–60 °C. The conductivity *σ* (mS/cm) was calculated from the obtained resistance *R* (Ω) according to the following equation.

*σ* (mS/cm) = *L*/(*S* × *R*) × 10^3^ (1)

where *L* (cm) is the distance between two electrodes, and *S* (cm^2^) is the cross-sectional area of the membrane obtained by multiplying the membrane thickness by the membrane width.

## 3. Experimental Results

The sPS-based membranes were characterized via UV-Vis, TGA, PGAA, optical microscopy, and WAXD prior to their investigation by SANS and the conductivity measurements. As reported elsewhere [22,23,24], membranes with different sulfonation and crystalline degrees were prepared, as shown by the PGAA and WAXD analysis, respectively. For the newly synthesized sPS-C70 composite membrane for the 1 wt% C70 fullerene content in the initial solution, a sulfonation degree of SD = 55% and a crystallinity of 29% were determined.

**Figure 1:**
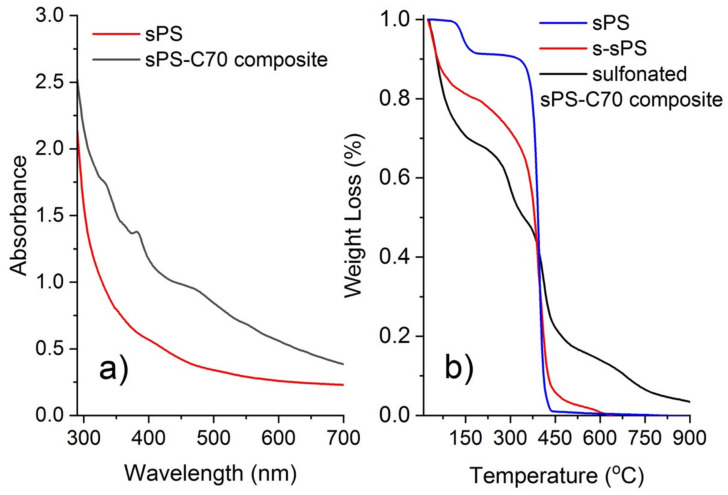
The UV-Vis spectra from the sPS–C70 composite membrane prior to sulfonation and a fullerene-free sPS film (**a**) and the thermogravimetric analysis (TGA) result from the sulfonated sPS–C70 composite membrane in parallel with results from the sPS and s-sPS films (**b**).

The incorporation of the fullerenes into the membrane was checked by UV-Vis before being exposed to the sulfonation reaction, and by TGS in the sulfonated state. The UV-Vis absorption spectrum of the composite membrane is shown in Figure 1a in parallel with the spectrum from an sPS film, and the TGA plots for three membranes, an sPS film, an s-sPS film, and the sulfonated sPS-C70 composite membrane, are displayed in Figure 1b. In the UV-Vis spectrum from the sPS film, one can observe that the characteristic absorption features of polymer occur below 300 nm while above this value the absorbance falls quickly off. Unlike this, the absorption spectrum from the composite membrane is significantly stronger above 300 nm, which indicates the incorporation of fullerenes into the membrane. The broad absorption band at around 470 nm, the absorption peak at around 380 nm, and the shoulder observed at around 330 nm are characteristic of the C70 absorption [31]. Thermal decomposition of the sPS membrane containing guest molecules (toluene) shows two stages in the TGA plot (Figure 1b): the loss of volatile guest molecules, from 100 to 200 °C, and the decomposition of the polymeric matrix, from 400 to about 600 °C. For the sulfonated membrane, an additional intermediate stage can be observed between these two decomposition processes, which is the degradation and decomposition of the sulfonate sites [1], which occurs gradually between 100 and 400°C. The higher temperature degradation process observed in the plot from the composite membrane arises from the decomposition of fullerenes [32]. These observations confirm that the C70 fullerenes are incorporated in the membrane.

**Figure 2:**
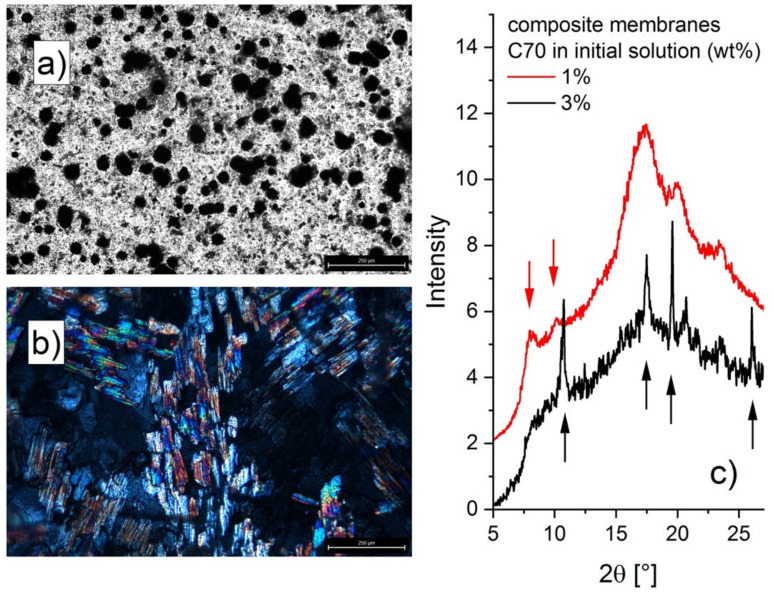
Micrographs (**a**,**b**) and wide-angle X-ray diffraction (WAXD) spectra (**c**) from sulfonated composite membranes obtained by casting of common sPS–C70 solution in toluene (for 1 wt% (**a**) and 3 wt% (**b**) fullerenes content in the initial solution) and subsequent sulfonation. C70 morphologies were observed by using bright field (**a**) and crossed polarizers (**b**) optical microscopy (the scale bar in panels (**a**,**b**) indicates 250 μm). The scattering patterns in panel (**c**) are shifted vertically for clarity. The black arrows in the panel (**c**) indicate the C70 crystalline reflections, as reported in [35], while the red arrows indicate the peaks characteristic to the δ-form of sPS clathrates [22].

The analysis of the sulfonated composite membrane under the optical microscope using the bright field option (Figure 2a) revealed C70 agglomerate particles with sizes of up to 25 µm, which are characterized by a very large polydispersity in size. The micrograph resembles that obtained from a C60-Nafion composite membrane [33,34] and proves the incorporation of fullerenes in the polymer matrix. The C70 agglomerates look similar in the composite membrane before sulfonation, which shows that the fullerenes are stably embedded in the membrane, even though the fullerenes are not chemically bound to the polymer. This confirms that syndiotactic polystyrene, similar to Nafion, can accommodate fullerenes in the amorphous regions [33]. It is noteworthy that at a higher C70 content in the initial polymer-fullerene solution (3 wt%), the formation of fullerene crystals in the membrane was observed by optical microscopy with crossed polarizers (Figure 2b). We can assume that the fullerenes incorporated in the syndiotactic polystyrene matrix are more effective in their role as radical scavengers if composite membranes are made with lower concentrations of fullerenes in the initial solution (up to 1 wt%), which leads to a better dispersion of fullerenes and their aggregates in the polymer matrix. A higher concentration of fullerenes in the initial solution leads to the formation of large crystals, which can also affect the mechanical stability of the membrane, as described in [16].

The δ-form of the sPS in the sulfonated composite membrane cast from the initial sPS-fullerene solution with 1 wt% C70 concentration was confirmed by the WAXD analysis (Figure 2c), as for the previously investigated membranes [22,23]. This indicates that the C70 fullerenes are mainly incorporated in the amorphous regions of the membrane, in a similar way as observed in the case of Nafion membranes [33]. As with the sPS-fullerene composite membranes [32] or the PMMA-C70 composite membranes [36] with low fullerene loading, in this case, no clear fullerene reflections were observed in the WAXD spectrum (the red diffraction pattern if Figure 2c). This may be indicative of a disordered positioning of neighbors around a given C70 molecule within the fullerene agglomerates. Although the C60 fullerenes could be incorporated as guests between the helices of the sPS chains of the crystalline regions, as reported in [32], there is no clear evidence of this effect in the WAXD spectra from our sPS-C70 composite membranes. Whether or not the C70 fullerenes may also be incorporated as guest molecules in the crystalline regions of the membrane is of no importance for this study, since, as mentioned in our earlier work, the neutron scattering of fullerenes, even in the aggregate form, is very weak in the investigated Q region in our SANS study, and would not affect the scattering from the crystalline and amorphous sPS morphologies. In addition, for isotropic membranes, the major contribution to the observable scattering would come from the functionalized and hydrated regions of the sPS polymer. Therefore, the possible presence of C70 fullerenes within the crystalline sPS lamellae is neglected in the current study. For a higher C70 content in the initial common polymer-fullerene solution, on the other hand, the reflections from the fullerene crystals are observed in the diffraction pattern (Figure 2c). The scattering characteristics typical for the sPS crystalline forms can no longer be distinguished. The optical microscopy and WAXD characterizations show that sPS-C70 composite membranes could play a role as PEM materials for a low fullerene load. In the case of the membranes that were produced by the casting of common polymer-fullerene solution with a C70 content of up to 1 wt%, the crystalline matrix of the sPS is still retained and therefore these membranes are suitable for such applications after their sulfonation, which does not appear to affect the fullerene dispersion in the membrane. At higher fullerene content, patches of large C70 crystals are observed, which also influence the formation of a robust crystalline polymer matrix by solution casting.

**Figure 3:**
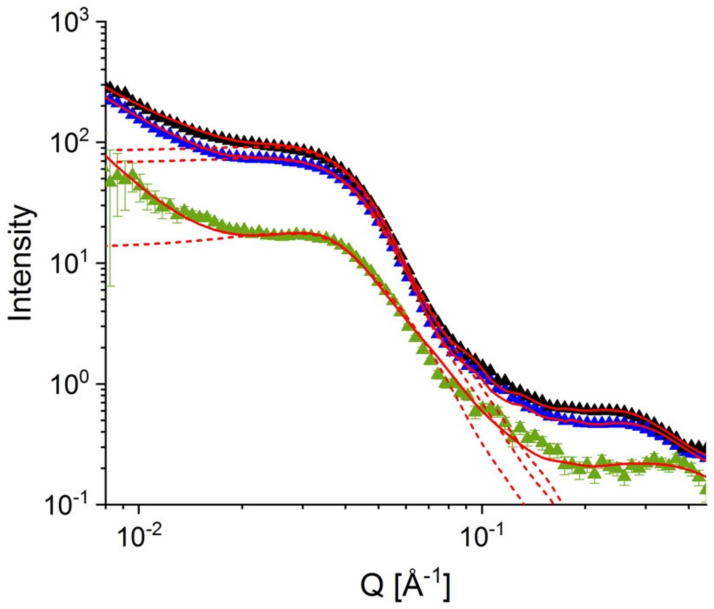
One-dimensional small-angle neutron scattering (SANS) patterns from the sulfonated composite membrane of sPS and C70 in dry state (green symbols) and hydrated state at 30 °C (black symbols) and 60 °C (blue symbols) for RH = 80%. The full red curves represent the fit of the experimental data with the model in Equation (2), while the dotted red curves depict the contribution of the correlated spherical domains to the global model.

Microstructural characterization by SANS was done on the sulfonated composite sPS-C70 membrane prepared by casting of solution with 1 wt% C70 fullerene content. SANS results on the composite membrane in the dry state at 30 °C (green symbols) and at RH = 80% and temperatures 30 °C (black symbols) and 60 °C (blue symbols) are shown in Figure 3. Since the membrane is obtained by the casting of an initial common solution of sPS and C70 in toluene, the scattering pattern is isotropically distributed on the two-dimensional neutron detector. Such scattering patterns can be analyzed by radially averaging the data over the entire detector and not over sectors as in the case of the uniaxially deformed membranes [22]. In all scattering patterns, three scattering features could be observed: (a) the power-law behavior in the small Q regime, where the typical upturn is observed due to the large-scale fractal character of the polymer film; (b) an intermediate Q regime between 0.01–0.1 Å^−1^, in which a broad feature occurs, which corresponds to the superposition of scatter signals from the randomly oriented crystalline regions (so-called “matrix knee”) and the dry sulfonated and hydrated sulfonated domains, respectively; and (c) the high Q regime (ca. 0.1–0.5 Å^−1^) in which the most characteristic feature is observed, namely the ionomer peak, which arises due to the correlation distance between the dry or hydrated ionic clusters in such polymer membranes.

Increased scattering intensity is observed from the membrane in the hydrated state compared to the dry sate, which is due to the water uptake at RH = 80%. As reported elsewhere [22,23], the hydration of sulfonated domains is changing drastically the scattering length density (SLD) of these domains due to the large difference between the SLD of the accumulated water (*ρ*_w_ = −0.56 × 10^10^ cm^−2^) compared to that of sulfonated sPS segments (*ρ*_sulf_ = 6.34 × 10^10^ cm^−2^). This leads to an increase in the scattering contrast between the sulfonated hydrated domains and the rest of the polymer matrix compared to the case of the dry membrane. At the same time, the position of the ionomer peak is shifted to the lower Q values when the membrane is hydrated, which is due to the increase in the correlation length between the ionic clusters as a consequence of the swelling of sulfonated domains when water is absorbed.

As already observed with similar membranes [24], the scattering intensity decreases slightly with increasing temperature at constant RH. This indicates a decrease in the scattering contrast, which can be due to slight water desorption. A very small shift in the ionomer peak position to higher Q values with increasing temperature at constant RH can only be assumed after inspection of the high Q region, and it seems to confirm what was previously the case for membranes with a lower degree of sulfonation and fullerenes content [24] when the effect was observed more clearly. This can be an indication of a small decrease in the correlation length between the ionic clusters with increasing temperature as a result of weak morphological changes due to the variation in the amount of absorbed water. The red lines in Figure 3 represent the model interpretation of the experimental data based on the model of scattering from correlated spherical sulfonated and hydrated domains, which was used in our previous studies [22,23,24]:

(2) $$I\left(Q\right)=\varphi \;\Delta \rho^{2}{\;V}_{d}\;P\left(Q\right)\;S\left(Q\right){+I}_{\mathrm{ion}}{+I}_{\mathrm{fract}}+\mathrm{Bckgd}$$

where P(Q) represents the form factor of the scattering domains, supposed spherical in shape, and S(Q) is the structure factor of the correlated domains, which appears for highly sulfonated membranes [23]. The form factor and the structure factor for such morphologies are described in detail in [25]. The contrast Δ*ρ* is the difference between the SLD of the sulfonated or hydrated domains and the rest of the polymer matrix. Usually, the factor (φΔ*ρ*^2^ V_d_) is called the “forward scattering” I_0_ from the scattering domains. The terms I_ion_ and I_fract_ represent the additional contribution at high Q, from the ionomer peak, which can be described by a Gaussian function, and at low Q, from the fractal behavior of the film, which can be described by a simple power-law term, P_3_Q^−3^, with P_3_ the power-law constant [25]. A constant background, Bckgd, which arises mostly from the incoherent scattering contribution from the film sample, is added as a final term of the model. The solid lines show the global fit of the scattering patterns while the dashed lines represent the scattering contribution of the correlated sulfonated or hydrated domains to the global fit [23]. The results of the fitting procedure show an increase in the radius of the sulfonated domains R_d_ by a factor of 1.28 due to hydration at 30 °C, while due to the increase of the temperature from 30 °C to 60 °C at the RH = 80%, a decrease factor in dimensions of 1.07 is obtained. This behavior also agrees fairly well with the shift in the position of the ionomeric peak observed in the high Q region. Since we cannot assess the scattering contribution of the crystalline areas when working with un-oriented membranes, in the following analysis, we will only refer to relative comparisons between the scattering patterns collected under different hydration and temperature conditions, which is a pretty good assumption since the change in the scattering contrast between different conditions is due to the water being absorbed only by the functionalized amorphous areas.

The neutron contrast in the dry membrane represents the difference in the SLD between the sulfonated and non-sulfonated polymer segments, $\Delta \rho_{d}^{s}$ = 0.34 × 10^10^ cm^−2^ [23], while in the hydrated membrane the contrast is produced due to difference in SLD between the non-sulfonated polymer segments and the hydrated domains, the latter being a partition between the SLD of the water molecules $\Delta \rho_{d}^{w}$ and sulfonated polymer segments $\Delta \rho_{d}^{s}$, each with its own volume fraction occupied in the hydrated domain, $\left(\varphi^{s}\Delta \rho_{d}^{s}+\varphi^{w}\Delta \rho_{d}^{w}\right)$. Thus, we can roughly calculate the volume fraction of water in the hydrated domains from the ratio between the forward scattering of the dry and hydrated sulfonated domains at 30 °C and RH = 80%, which is about $\varphi^{w}$ = 15%. Considering the sulfonation degree of the membrane, we can estimate a volume fraction of 8.5% at 30 °C for RH = 80%, which is occupied by water in the membrane. In the same way, we can estimate that with a temperature increase from 30 °C to 60 °C at an RH = 80% the water volume fraction in the membrane decreases by 11%, which indicates low water desorption with increasing temperature at constant RH. It should be noted that in the above calculation, we neglected the presence of C70 fullerenes incorporated by the membrane. As discussed elsewhere [23], the contribution of the fullerenes, even in their aggregation state, to the total scattering from the membrane is negligible under the conditions of the current study. However, the fullerene aggregates can change the conditions for rationalizing the volume fraction occupied by different species in the membrane, so the numbers from the above calculations should be considered as a rough estimate.

The proton conductivity of the membranes depends on the amount of water absorbed, which depends on the degree of sulfonation of the membranes. In Table 1, we report the results of the proton conductivity measurements on the sulfonated C70-sPS composite membrane (SD = 55%) under various humidity and temperature conditions. The results are shown in parallel with the observations made on a sulfonated uniaxially deformed sPS membrane (SD = 45%) in liquid water, previously reported in [24]. The composite membrane is characterized by a higher proton conductivity than the fullerene-free membrane at room temperature, which is due to its higher degree of sulfonation. However, although both membranes have a higher proton conductivity at high temperature in liquid water than at room temperature, the increase in proton conductivity of the composite membrane at 80 ° C is much more significant than that of the fullerene-free membrane. Here, we can speculate that this is due to the presence of the fullerenes built into the amorphous area of the membrane. In the vapor phase, the proton conductivity of the composite membrane, although it shows much lower values than in the liquid phase, still increases when the relative humidity, that is, the amount of water absorbed by the membrane, is increased. At RH = 80%, however, the proton conductivity decreases drastically with increasing temperature from 30 °C to 60 °C, contrary to the expected Arrhenius behavior. From this observation, one can directly infer the possible interruption of the water paths with increasing temperature for the membrane hydrated from the vapor phase. Furthermore, the activation energy for proton conduction in the two membranes in liquid water hydration state was evaluated from the Arrhenius dependence of conductivity on temperature.

(3) $$\sigma =A\;exp\left(-\frac{E_{a}}{\mathrm{RT}}\right)$$

where σ, A, E_a_, R, and T denote the proton conductivity, pre-exponential factor (mS cm^−1^), activation energy for proton conduction, ideal gas constant (8.314 J K^−1^ mol^−1^), and absolute temperature (K), respectively. The activation energy E_a_ that was estimated from the slope of the linear plot of ln σ against 1000/T is reported in Table 1.

## 4. Discussion

The proton conductivity of the sPS-based sulfonated membranes that we have produced and characterized in this study is comparable to that of the sPS sulfonated membranes reported in [17,37]. The proton conductivity measured when the membrane is hydrated from liquid water differs between the uniaxially elongated sPS film and the sPS–C70 composite membrane, as expected due to the difference in the degree of sulfonation (Table 1). In addition, the proton conductivity shows an Arrhenius behavior for both membranes in this hydration condition. The drastic increase in proton conductivity observed in the composite membrane at 80 °C by more than twice the value observed at 30 °C differs significantly from the behavior of the fullerene-free sPS membrane, which may be attributed to the incorporation of fullerenes into the composite membrane. According to [33], possibilities of additional water trapped in the interface between the fullerene aggregates and the polymer domain or morphological changes of polymer caused by the introduction of fullerenes into the polymer matrix may be considered in the case of composite membranes of Nafion and C60 fullerenes. The activation energy for proton conduction (E_a_) represents the minimum energy required for the proton transport from one free site to another. The E_a_ for the s-sPS membrane is similar to that in Nafion [38]. The composite membrane exhibits higher E_a_, which means that proton migration requires more energy. This observation, together with the higher conductivity measured in such membranes, indicates that a complex proton transport mechanism takes place in the sPS membranes that incorporate fullerenes. However, the detailed study of the proton transport mechanism in terms of either the Grotthuss (hopping) or the vehicle (diffusion) mechanism [39] requires a more complex characterization and the analysis of a number of membranes made with different compositions and treated under different humidity and temperature conditions, which is beyond the scope of the current manuscript. At this level of investigation, we can only speculate that in the case of the sPS–C70 composite membranes, the addition of fullerenes influences the development of the water paths and the proton transport mechanism in the hydrophilic regions of the polymer, possibly also through an effect on the conformation of the amorphous polymer segments. The latter can be tested when examining the composite membranes and fullerene-free membranes at different temperatures using quasi-elastic neutron scattering (QENS), which provides information about the segment dynamics of the amorphous polymer chains under different conditions, an investigation that is currently in progress on our membranes.

The proton conductivity of the composite membrane increases with the RH at constant temperature (30 °C): the more water the membrane absorbs, the hydrophilic domains swell and percolate, which facilitates proton conduction. At RH = 80% and 30 °C the sPS-C70 composite membrane shows a proton conductivity comparable to that of the Nafion 117 in similar conditions [40]. One should note that at RH = 50% the value of conductivity shown by the sPS-C70 membrane is very low and much lower than that observed in the case of Nafion 117. We may draw a first qualitative conclusion regarding the possible different formation and evolution of hydration pathways in the two materials. A drastic decrease in the proton conductivity of the sPS-C70 composite membrane was observed with increasing temperature at constant RH = 80% (Table 1). In the case of thermally treated Nafion 117, a slight decrease in proton conductivity was observed with increasing temperature between 20 °C and 45 °C due to water loss from the membrane, while a further temperature increase, above 45 °C, led to an increase in proton conductivity with temperature. The decrease in conductivity was attributed to structural changes caused by the heat treatment of the membranes prior to the measurement. However, the decrease in proton conductivity observed for the heat-treated Nafion 117 was modest.

**Figure 4:**
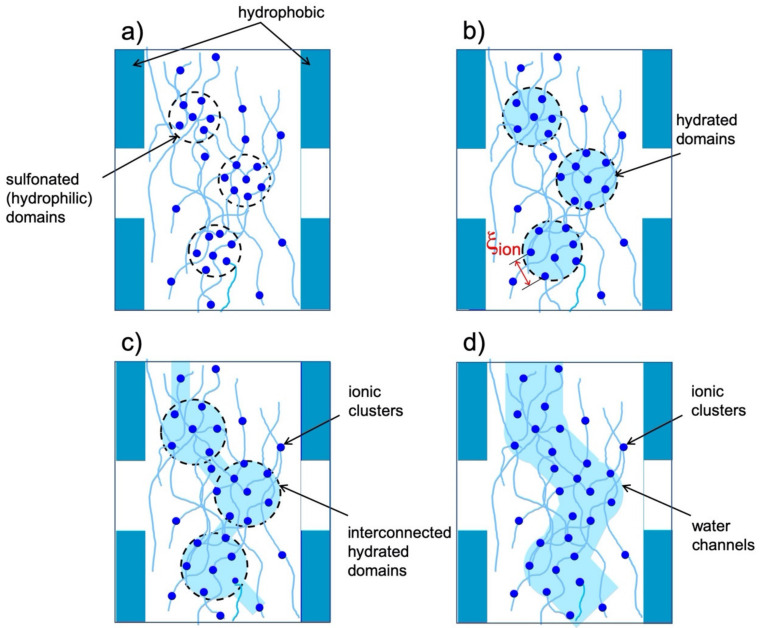
Proposed morphological descriptions for sPS based hydrophilic membranes emerged from the SANS investigation of membranes in different hydration and temperature conditions: clusters of sulfonated domains in dry state (**a**) are hydrated following the water sorption by the membrane, giving rise to hydrated clusters (**b**), which grow in size and become interconnected in increasing the hydration of membrane (**c**). The membrane becomes conductive in the state corresponding to the morphology shown in panel (**c**). At high hydration levels or when the membrane is immersed in liquid water, the interconnected water clusters evolve in cylindrical water channels (**d**). The rising of temperature on the membrane in the morphological state depicted in panel (**d**) leads to interruption in the interconnectivity of water domains, which affects the membrane conductivity.

The observed drastic drop in the proton conductivity of the sPS-C70 composite membrane at 60 °C compared to the value measured at 30 °C should be attributed to a morphological change in the hydrated domains rather than to the water desorption in increasing temperature. Based on the interpretation of the forward scattering of the hydrated domains provided by the SANS data, only a small decrease in water content of about 11% is observed when the temperature on the membrane is increased from 30 °C to 60 °C while maintaining the RH = 80%. To relate this observation with the observed large drop in proton conductivity, a structural idealization is proposed in Figure 4 for the formation and development of the hydrated domains and water paths in this type of membrane. In this model, only the formation and development of the morphology in the bulk amorphous region as a result of sulfonation and hydration processes is discussed. In addition, for the sake of simplicity of the model, which is based only on SANS and proton conductivity observations, the presence of C70 fullerenes is neglected.

Figure 4a shows the schematic view of the proposed morphology of the sulfonated amorphous regions: sulfonated domains, in which the correlation between ionic clusters takes place over the distance ξ_ion_, are indicated by dashed lines. The correlation between the ionic clusters leads to the appearance of the ionomer peak, which is observed in the scattering pattern from the membrane even in the dry state. The sulfonated domains are characterized by a neutron SLD that differs from that of the non-sulfonated or crystalline polymer domains (see the discussion in [22,23,24]), which creates a SANS contrast that leads to the appearance of the broad scattering feature that can be observed in the scattering pattern at the intermediate Q range. During the hydration process, first water molecules ionize and bind to the sulfonic acid groups via hydrogen bonds, which enables the bound counterions to dissociate. As more water is absorbed, phase separation is promoted and hydrophilic ion-rich domains are formed, as shown in Figure 4b. The correlation length between the ionic clusters ξ_ion_ increases as a consequence of the swelling of the domains, hence the shift in the ionomer peak position to lower Q values as the membrane hydration level increases. At higher RHs, additional water molecules cause further growth and connectivity of the hydrophilic domains, as shown in Figure 4c. The dissociated protons, which form ion-pairs with water, are transported through the interconnected hydrated domains, promoting membrane conductivity. A further increase of hydration level leads eventually to bulk-like water regions (channels) where the water molecules move freely (Figure 4c), which is the case of the membranes immersed in water. Water channels were evidenced by cryo-TEM in the uni-axially deformed s-sPS membranes in this state and characterized by contrast variation SANS analysis [22].

The evolution of the morphology of the composite sPS-C70 sulfonated membrane in increasing RH and temperature during our SANS and proton conductivity measurements corresponds to the stages depicted in Figure 4a–c. Given the low water desorption in increasing temperature while maintaining the same RH, as it was revealed by SANS, the observed drastic decrease in proton conductivity at RH = 80% when the temperature on the membrane is raised from 30 °C to 60 °C can only be assigned to interruptions occurring in the water connections between hydrated domains shown in Figure 4c. While the amount of water adsorbed by the membrane remains relatively high, the conduction paths are interrupted, thus the proton conductivity decreases significantly. Assuming the same pre-exponential factor determined from the linear variation in conductivity as a function of inverse temperature (Equation (3)), an activation energy E_a_ = 22.1 kJ mol^−1^ for the composite membrane at RH = 80% and 30 °C is roughly estimated, which indicates that small ion clusters are formed under these conditions, whereby the proton migration requires more energy. It is therefore expected that the connectivity between such clusters is easily broken with increasing temperature, while the RH is kept constant, with consequences for proton conduction.

These observations support the water cluster model, which may be characteristic of ionomer membranes based on syndiotactic-polystyrene. The behavior of the membrane conductivity with the variation of RH and temperature can only be explained if an organization of the hydrophilic domains in interconnected hydrated clusters is considered [41], which swell to form cylindrical channels as a result of the continuous water uptake through the membrane. In Table 1 we have specified the morphology assigned to the hydrated domains in the sPS-based membranes under different conditions.

## 5. Conclusions

Small-angle neutron scattering investigation and proton conductivity measurements have been carried out on a sulfonated composite membrane of syndiotactic-polystyrene and C70 fullerenes in various hydration and temperature conditions. In comparison with earlier reported similar measurements on syndiotactic-polystyrene-based membranes with variable sulfonation degree and crystallinity, a clear image of the formation and evolution of the hydrated morphologies in such materials in different hydration/temperature conditions could be achieved. Water clusters form and evolve in increasing the relative humidity RH and become interconnected, leading to proton conductivity in such membranes. At high hydration levels or in liquid water the clusters evolve in cylindrical channels through which the water moves freely, which confers the membranes a high proton conductivity. The proton conductivity of the membrane increases by increasing the RH at a constant low temperature (30 °C), while in increasing temperature (60 °C) it drops drastically. Based on the observation that only minor water desorption at 60 °C was estimated from the SANS experiments, we conclude that the water clusters, though still present in the membrane, are losing their interconnectivity in increasing temperature, which explains the observed proton conductivity behavior.

## Figures and Tables

**Table 1:** The proton conductivity shown by two sPS-based membranes, one uni-axially deformed s-sPS film and one sulfonated composite membrane of sPS and C70, with different sulfonation degrees, as measured in different humidity and temperature conditions, and the corresponding morphology of the hydrated domains, as evaluated from the SANS data reported in this work or from combined SANS and cryo-TEM (cryogenic transmission electron microscopy) observations reported in previous publications.

Sample	Hydration/Temperature	σ (mS cm^−1^)	Morphology ^1^	E_a_ (kJ mol^−1^)
s-sPS uniaxially	Liquid water, 30 °C	128	Cylindrical channels	3.95
deformed	Liquid water, 80 °C	160	Cylindrical channels	
SD = 45%				
	Liquid water, 30 °C	180	Cylindrical channels	
sPS-C70	Liquid water, 80 °C	450	Cylindrical channels	16.4
sulfonated composite	RH = 50%, 30 °C	1.5	Spherical clusters, partially interconnected	
SD = 55%	RH = 70%, 30 °C	10	Spherical clusters, interconnected	
	RH = 80%, 30 °C	19	Spherical clusters, interconnected	
	RH = 80%, 60 °C	1.3	Spherical clusters, partially interconnected	

^1^ from SANS ([22,23,24], current work) and cryo-TEM [22] characterization.

## Data Availability

The data presented in this study are available on request from the corresponding author.
